# Eye movement desensitisation and reprocessing (EMDR) treatment associated with parent management training (PMT) for the acute symptoms in a patient with PANDAS syndrome: a case report

**DOI:** 10.1186/s13052-019-0667-1

**Published:** 2019-06-26

**Authors:** Cristiana A. Guido, Anna Maria Zicari, Marzia Duse, Alberto Spalice

**Affiliations:** 1 Department of Pediatrics, Child Neurology Division, Sapienza University of Rome, 00161 Rome, Italy; 2 Department of Pediatrics, Pediatric Division, Sapienza University of Rome, 00161 Rome, Italy

**Keywords:** Eye movement desensitisation and reprocessing treatment, PANDAS/PANS syndrome, Parent training, Adverse childhood experiences

## Abstract

**Background:**

The purpose of this report was to present the results of eye movement desensitisation and reprocessing (EMDR) therapy associated with parent management training (PMT) in a child with paediatric autoimmune neuropsychiatric disorder associated with streptococcus (PANDAS), who had previously only been treated with pharmacological treatment.

**Case presentation:**

The case concerns an 11-year-old boy who presented with simple and complex vocal tics, motor tics, obsessive-compulsive traits and irritability from the age of 6 years, in addition to a positive result for streptococcal infection. The course of symptoms followed a relapsing-remitting trend with acute phases that were contingent on the infectious episodes.

**Conclusions:**

Following eight sessions of EMDR, preceded by training sessions with the parents, the child showed a significant reduction in symptoms and disappearance of the exacerbation. These results indicate the possibility of improving the treatment outcomes of patients with PANDAS by a combined approach using both antibiotic and EMDR therapies.

## Background

Paediatric autoimmune neuropsychiatric disorder associated with streptococcus (PANDAS) is a disorder characterised by the acute onset of obsessive-compulsive disorder (OCD) and/or tics, often accompanied by attention deficit/hyperactivity, separation anxiety, oppositional behaviours and emotional lability [1]. This is often accompanied by personality changes, cognitive disturbances, motor abnormalities, sensory sensitivity, behavioural regression, and, occasionally, psychosis [2, 3]. PANDAS patients with tics experience greater impacts at home and school, associated with poor academic performance, impaired writing, impaired visual and spatial memory and low self-esteem [4]. The pathogenesis of PANDAS syndrome is hypothesised to be related to group A streptococcal (GAS) infection via molecular mimicry, whereby streptococcal antibodies target brain proteins with a similar epitopic structure to that of GAS [5]. PANDAS syndrome exhibits immunological similarities to Sydenham’s chorea (SC), a classic infection-triggered autoimmune disorder that also presents a high degree of OCD comorbidity [1]. However, unlike SC, evidence for autoantibody-mediated reactions in PANDAS has been less conclusive [6, 7]. A newer iteration of acute-onset neuropsychiatric symptoms that are not dependent upon an association with GAS has been termed “pediatric acute-onset neuropsychiatric syndrome” (PANS) [8]. Given the dramatic phenotype and the potential for long-term neuropsychiatric sequelae, PANS may represent a subtype of autoimmune encephalopathy that primarily affects psychiatric symptom domains rather than the neurological symptom domains typical of other encephalopathies [9, 10].

According to the most recent guidelines [11], elective evidence-based therapies for the treatment of PANDAS/PANS include Cognitive Behavioural Therapy (CBT), parent training and drug therapy. A small study of intensive CBT with family involvement found that the symptoms of OCD improved in PANDAS patients during an acute episode, and these benefits remained even following relapse [12]. The application of parent management techniques (PMT) include encouraging families not to participate in behaviours associated with the child’s avoidance and ritual behaviours, positive reinforcement of desired behaviours, establishing clear limits, expectations and consequences and creating rewards. Parent management techniques are particularly useful when patients with PANS are not ready or willing to use CBT [13].

Eye movement desensitisation and reprocessing (EMDR) therapy has been used to treat various psychiatric disorders associated with Post Traumatic Stress Disorder (PTSD), but has never been utilised in patients with PANDAS syndrome. EMDR can be considered a psychotherapeutic approach which focuses on how information is stored in memory. Eye movement desensitization and reprocessing (EMDR) is an empirically validated psychotherapy approach that medical personnel can employ to treat the sequelae of psychological trauma and other negative life experiences. Its ability to rapidly treat unprocessed memories of these adverse experiences has important implications for the medical community, as they appear to be the foundation for an array of clinical symptoms. Clinical applications of EMDR include a wide variety of psychological problems affecting patients and family members, as well as stress-induced physical disorders. EMDR therapy is an eight-phase treatment approach composed of standardized protocols and procedures. The eight phases and three-pronged protocol facilitate a comprehensive evaluation of the clinical picture, client preparation, and processing of a) past events that set the foundation for pathology, b) current disturbing situations, and c) future challenges. One of the components used during the reprocessing phases is composed of dual attention stimuli in the form of bilateral eye movements, taps, or tones. The explanatory model underlying these considerations is the Adaptive Information Processing model, (AIP) in which previously stored dysfunctionally without proper assimilation within of a wider adaptive network [14]. The (AIP) model posits that, except for symptoms caused by organic deficits,toxicity, or injury, the primary foundations of mental health disorders are unprocessed memories of earlier life experiences. However, as indicated in the AIP model, a wide range of adverse life experiences can also be stored in a dysfunctional manner, providing the basis for diverse symptomology that include negative affective, cognitive, and somatic responses. Sufficient processing of those accessed memories within the standard three-pronged EMDR therapy protocol brings about adaptive resolution and functioning. It is conjectured that processing the targeted experiences transfers them from implicit and episodic.

memory to explicit and semantic memory systems. The originally experienced negative emotions, physical sensations, and beliefs are altered as the targeted memory is integrated with more adaptive information. What is useful is learned and stored with appropriate affective, somatic, and cognitive concomitants. Consequently, the disturbing life experience becomes a source of strength and resilience [14].

According to this model, positive and negative attributions are fuelled by experiences stored in memory; personality traits are considered characteristic responses that are based on stored experiences; and symptoms that are not entirely organic in nature are based on these stored experiences. EMDR works on the present and not on the past. Processing of the memory is triggered from the material physically present in the patient’s associative networks, a procedure which favours the development of new, more adaptive networks that connect the dysfunctionally stored material to positive and adaptive networks present in the patient [14].

Herein, we report the case of a patient with PANDAS who showed a marked reduction in tics and OCD behaviour following EMDR therapy.

## Case presentation

An 11-year old boy came to our attention, presenting with acute onset of simple and complex motor tics, oral tics, obsessions and compulsions (fear of contamination, aggressive behaviour, superstitions, repetitive compulsions, religious compulsions, etc.), irritability and anxiety. A deterioration in writing (micrographia) was also observed.

The boy had been delivered at term after an uneventful pregnancy, and developmental milestones were reached at normal ages. Birth weight was 3.200 g emotional and social development within normal range. At 6 years of age, the parents noticed a subtle beginning of tic disorders, which slowly disappeared.

In regard to family history, the patient’s father suffered from OCD and tics during childhood, while his mother suffered from thyroid cancer, Hashimoto’s thyroiditis and depression.

The boy suffered from recurrent familial streptococcal infection, celiac disease was also diagnosed.

Due to elevated Antistreptococcal (ASO) (650 UI nv 0–200) and antiDNAse titres (950 UI nv 0–300), associated with positive swab test for streptococcal infection during exarcebation together with the abrupt onset of clinical presentation, a diagnosis of PANDAS syndrome was made. The boy underwent treatment with antibiotics (oral amoxicillin), followed by prophylaxis with penicillin (1.200,000 UI every 21 days). From 2016 until 2017, the patient received prophylactic treatment, and an adenotonsillectomy was performed in 2016. The patient’s clinical condition was stable, with slight improvement in accordance with the relapsing-remitting course.

During the period of observation, the boy does not show a complete remission of the symptoms, that worsened during infection sometimes associated with positive swab test for streptococcocal infection; also ASO and antiDNAse titres remain elevated (530 UI and 900 UI respectively).

The boy underwent a full neuropsychological evaluation, including the Child Behavior Checklist (CBCL)(6–18 years), Conners’ Parent Rating Scales Long Version (CPRS-R:L) Yale Global Tic Severity Scale (YGTSS) and Child Yale-Brown Obsession and Compulsion Scale (CY-BOCS).

The results of EEG and MRI scans were normal, with no basal ganglia alterations and a normal cortical structure.

Due to poor response to prophylaxis with penicillin, PMT was performed with the parents and child, followed by individual sets of EMDR.

The work with EDMR was initially focused on acute episodes related to tics. The installation of resources favoured the transition from an external to internal focus, with the creation of new coping strategies that were more adaptive than previous ones.

Before EDMR treatment, the boy was assessed according to the YGTSS and CY-BOCS, and then re-examined with the same scales after a 6-month follow-up period. The scores are presented in Table 1.

**Table 1:** Yale Global Tic Severity Scale (YGTSS) and Child Yale-Brown Obsession and Compulsion Scale (CY-BOCS) score before and after eye movement desensitisation and reprocessing (EMDR) treatment

YGTSS	Pre-treatment T score	Post-treatment T score
*Impairment (0–50)*	30	0
*Total tic severity score (motor tic severity + vocal tic severity; 0–50)*	26	12
*Total YGTSS score + impairment (0–100)*	56	12
Severity grade	Moderate	None
CY-BOCS		
*Obsessive*	5	0
*Compulsive*	12	8
*Total score (compulsion + obsession)*	17	8
Severity grade	Moderate	Minimal

The boy showed great improvement in motor and vocal tics following treatment, in addition to a marked reduction in obsessions and compulsions. At present, after 2 years of follow-up, he has completed EMDR treatment with great clinical improvement. No more tics or obsessive compulsion disorders were noticed. He is not currently receiving prophylactic treatment. In addition, ASO and antiDNAse titres are within normal ranges.

### Eye movement desensitisation and reprocessing treatment (EMDR) protocol

In the present case, treatment with EMDR was proposed, with the aim of reforming the patient’s unfavourable experiences and improving his coping strategies.

The EMDR procedure consists of eight stages. The 8 stages consist of:

- Phase 1 - *History taking*: obtain background information, identify suitability for EMDR treatment.
- and identify processing targets from events in client’s life according to standardized three-pronged protocol;
- Phase 2 - *Preparation*: prepare appropriate clients for EMDR processing of targets;
- Phase 3 - *Assessment*: access the target for EMDR processing by stimulating primary aspects of the memory;
- Phase 4 - *Desensitization:* process experiences toward an adaptive resolution (no distress);
- Phase 5 - *Installation:* Increase connections to positive cognitive networks;
- Phase 6 - *Body Scan*: Complete processing of any residual disturbance associated with the target;
- Phase 7 - *Closure*: Ensure client stability at the completion of an EMDR session and between sessions;
- Phase 8 - *Reassessment*: Ensure maintenance of therapeutic outcomes and stability of client. (Shapiro 2014).

After taking the patient’s history, explaining EMDR and identifying the most distressing seizure-related memories (i.e., target selection), the target image(s) are processed consecutively. The therapist asks the patient to keep the disturbing target memory and aspects related to it in mind, while simultaneously performing a distractive task introduced by the therapist. The child is asked to follow the therapist’s finger, making saccadic movements at a rate of about two stimuli per second for approximately 30 s. In the case of problems with eye movements, auditory bilateral stimulation or tactile stimulation is used. The child is then asked to briefly report what comes to mind (associations). The procedure is repeated until the original target is no longer disturbing, and dysfunctional cognitions regarding the trauma have become functional [15].

Upon completion of the eight phases, we proceeded with the Installation of the Resources and with the Future Model, where the patient is asked to project themselves into the future problematic situations and imagine using the new resources installed.

### Parent management training (PMT)

Parent management training is a training and information course (training) which is focussed on the parents. In recent years, our clinical knowledge concerning behavioural disorders during development has increased, and interventions have moved toward integrated clinical interventions that involve the parent(s) as an active part of the treatment. Parent training aims to establish an active collaboration of parents through appropriate training and training procedures. It is based on the theory of social learning, and has been implemented for uncooperative, oppositional children with behavioural disorders or attention-deficit hyperactivity disorder [16]. Currently, PMT is suggested as one of a large number of treatments that can be applied during the child’s development.

Parent training is applicable in all circumstances in which parents have to face an educational task with problematic children. The general objectives of the intervention involve informing parents about the manifestations of the disorder, the symptoms, and the vicious circuits that maintain them, thereby promoting acceptance of the diagnosis and collaboration between parent and child.

Parents are trained through the teaching of educational methods on functional analysis of behaviour in order to increase the educational skills best suited to the child. The aim is to improve the style of family communication, especially between parents and children.

## Discussion

To our knowledge, EMDR therapy is utilised in children and adolescents, and had never been utilised in patients with PANDAS/PANS syndrome [17].

In our patient, EMDR clearly reduced total tic severity and total YGTSS score, as well as the severity grade, as shown in Table 1. At the same time, the patient showed a marked reduction in obsessions and compulsions, assessed by the CY-BOCS, as well as the severity grade (Table 1). In particular, the score for compulsive disorder reduced from 12 to 8, while obsessive disorder completely disappeared (5 to 0). After 6 months of follow-up, his clinical condition remains stable and good.

One of the hypotheses for how EMDR works is based on the concept of ACE, which represents the number of negative experiences of children. Cognitive and neuroscience researchers have examined possible mechanisms that might explain the negative consequences of ACE on adult health [18] Adverse childhood experiences can alter the structural development of neural networks and the biochemistry of neuroendocrine systems [19], which may have long-term effects on the body, including speeding up the processes of disease and aging and compromising immune systems [20]. These mediators of the stress response promote adaptation in the aftermath of acute stress, and also contribute to allostatic overload, the wear and tear on the body and brain that result from being ‘stressed out’. This conceptual framework has created a need to determine how to improve the efficiency of the adaptive response to stressors while minimising overactivity of the same systems, as such overactivity results in many of the common diseases of modern life. This framework has also helped to demystify the biology of stress by emphasising both the protective and damaging effects of the body’s attempts to cope with stressors [21].

In the present case, following the diagnosis of PANDAS/PANS, the boy received from the parents a long term overcontrol on simple actions that limited the boy on the natural activities (playing, eating, etc). This situation may depicts an emotional abuse or a maladaptive response that it is defined as nonaccidental verbal or symbolic acts by a child’s parent or caregiver that result or have reasonable potential to result in significant psychological harm to the child. This condition may be considered in our patient an adverse childhood experience.

On the other hands, the unpredictable nature of exacerbation of symptoms produces an increase in the external locus and a reduction in the sense of self-efficacy with regard to controlling the child’s symptoms. The parents can display overprotective behaviour and control over their child, reducing autonomy and increasing the feeling of internal discomfort. PMT may have obtained a more flexibile educational style that may reflect also on the tics reduction.

It has been demonstrated that long periods of stress may stimulate our hortosympatic system, leading to elevated cortisol and subsequent elevation of inflammatory cytokines [22]. In animal models of PANDAS, an elevation in interleukin 17 has been reported, highlighting its potential use as a biomarker and as a potential target for new therapies. This observation has led to speculation that EMDR therapy may play a role in the regulation of this kind of neuroinflammation.

In our patient also therapeutic use of penicillin and tonsillectomy was performed without definitive results as previously indicated [23].

Parent management training may be a valuable tool for helping parents of children with neuropsychiatric conditions and for parents of PANDAS patients. It has been hypothesised that this method could also be applied in parents that face stressful and difficult family situations. In our case, PMT appeared to help the parents avoid stressful situations that could have enhanced the patient’s tics and OCD.

## Conclusion

Our results suggest that EMDR may be utilised in patients with PANDAS/PANS syndrome, together with PMT. Additional studies investigating the application of these therapies in a larger population are necessary.

## Data Availability

All data generated or analysed during this study are included in this published article.

## Abbreviations

ASO: Antistreptococcal
CBCL (6–18 years): Child Behavior Checklist
CBT: Cognitive Behavioural Therapy
CPRS-R:L: Conners’ Parent Rating Scales Long Version
CY-BOCS: Child Yale-Brown Obsession and Compulsion Scale
EMDR treatment: Eye movement desensitisation and reprocessing
GAS: Group A streptococcal
OCD: Obsessive-compulsive disorder
PANDAS: Paediatric autoimmune neuropsychiatric disorder associated with streptococcus
PANS: Pediatric acute-onset neuropsychiatric syndrome
PMT: Parent management training
YGTSS: Yale Global Tic Severity Scale
